# Supplementary material for: Genetic dissection of cyclic pyranopterin monophosphate biosynthesis in plant mitochondria
Source: Biochem J. 2018 Jan 31;475(2):495–509. doi: 10.1042/BCJ20170559 (PMC5791162; doi:10.1042/BCJ20170559)
Supplement: Supplementary Figures and Table [file BCJ-475-495-s1.pdf]

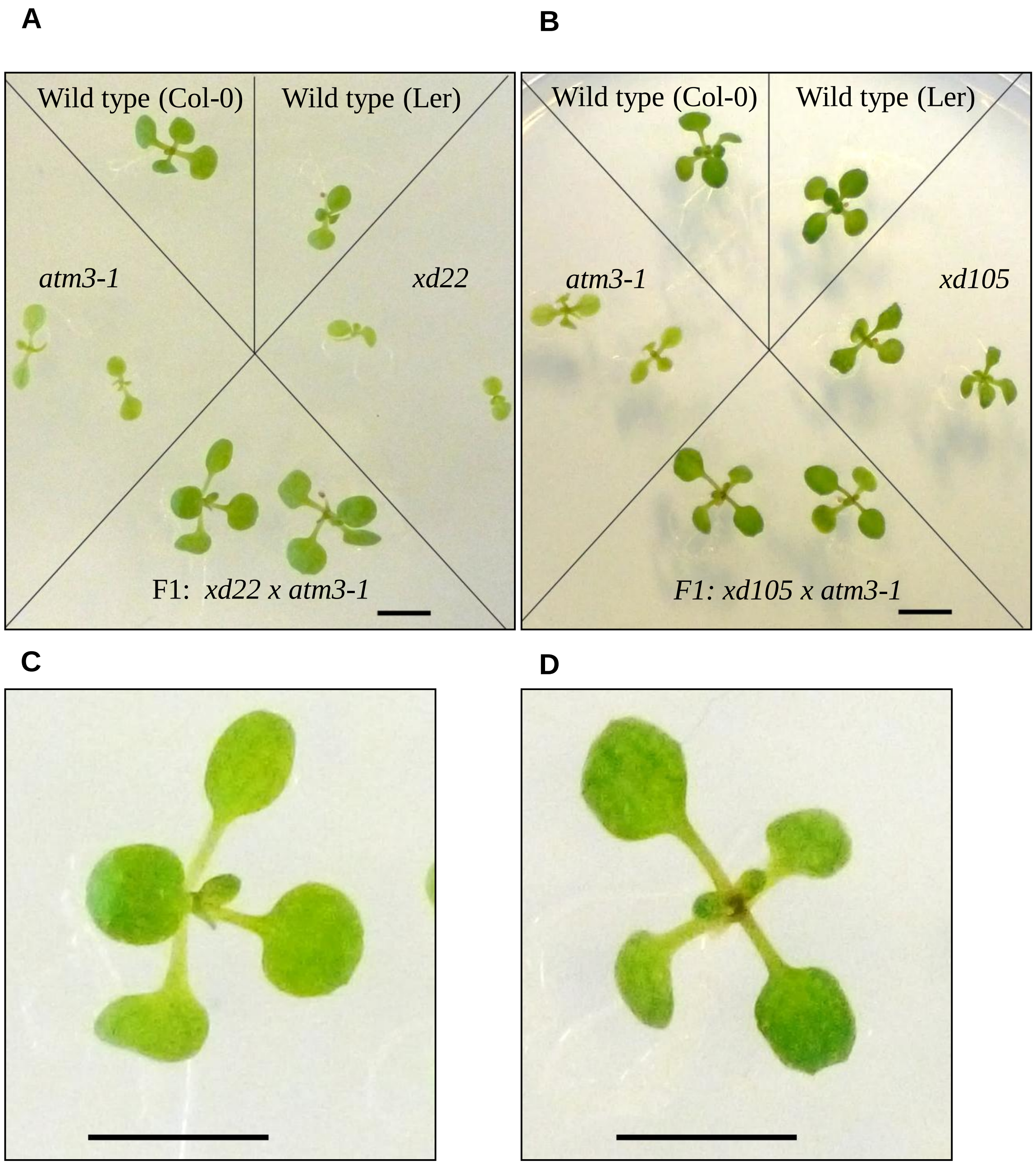

Supplementary Figure S1. Growth of parental and F1 plants from *xd22* x *atm3-1* and *xd105* x *atm3-1* crosses. Plants were grown for 11 days (A) or 15 days (B) on ½ MS-agar plates. *atm3-1* and *atm3-4* are in the Col-0 background, *xd22* and *xd105* are in the Ler background. Close-up of an F1 plant from *xd22* x *atm3-1* (C) and *xd105* x *atm3-1* (D). Scale bar is 0.5 cm.

A B

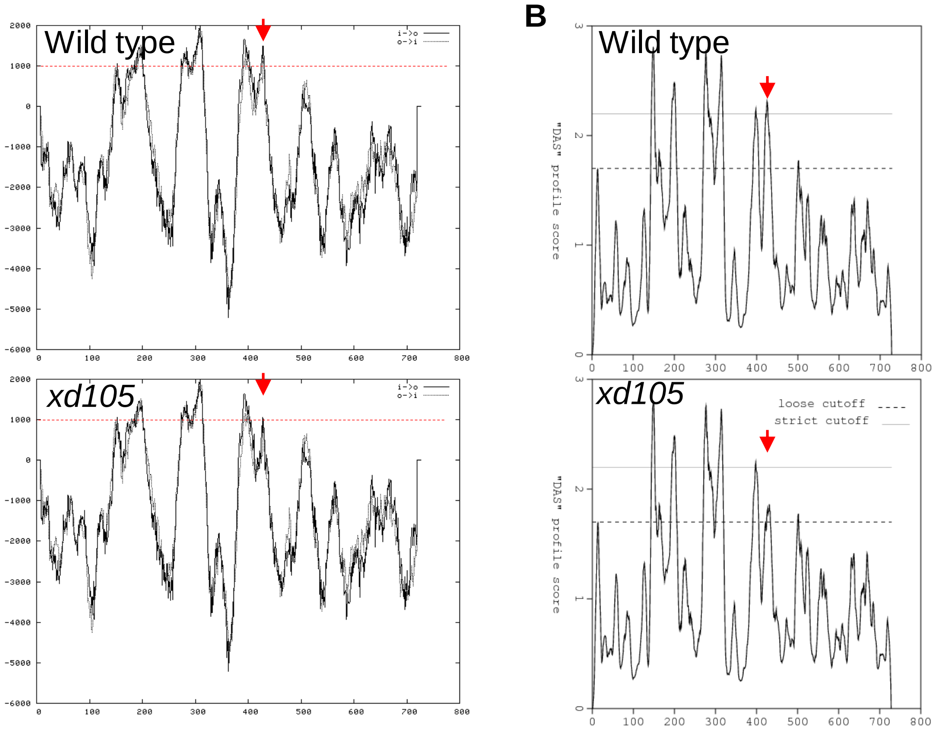

C

```
WT
seq   AMVLCSQGIV NGQMTVGDLV MVNGLLFQLS LPLNFLGSVY RETIQSLVDM   450
pred  HHHHoooooo ooooooooHH HHHHHHHHHH HHHHHHHHHH iiiiiiiiii

seq   KSMFQLLEEK SDITNTSDAK PLVLKGGNIE FENVHFSYLP ERKILDGISF   500
pred  iiiiiIIIII IIIIIIIIII IIIIIIIIii iiiiiiiiii iiiHHHHHHH

seq   VVPAGKSVAI VGTSGSGKST ILRMLFRFFD TDSGNIRIDG QDIKEVRLDS   550
pred  HHHHHHHHHH HHHooooooo ooooooooOO OOOOOOOOOO OOOOOOOOOO

xd105
seq   AMVLCSQGIV NGQMTVGDLV MVNELLFQLS LPLNFLGSVY RETIQSLVDM   450
pred  HHHHoooooo ooooooooOO OOOOOOOOOO OOOOOOOOOO OOOOOOOOOO

seq   KSMFQLLEEK SDITNTSDAK PLVLKGGNIE FENVHFSYLP ERKILDGISF   500
pred  OOOOOOOOOO OOOOOOOOOO OOOOOOOOOO OOOOOOOOOO OOOOOOOOOO

seq   VVPAGKSVAI VGTSGSGKST ILRMLFRFFD TDSGNIRIDG QDIKEVRLDS   550
pred  OOOOOOOOOO OOOOOOOOOO OOOOOOOOOO OOOOOOOOOO OOOOOOOOOO
```

Supplementary Figure S2. Modelling of structural changes caused by Glycine 424 into Glutamate (G424>E) in ATM3

A. Secondary structure prediction using TMPred (www.ch.embnet.org/cgi-bin/TMPRED_form_parser). The red arrow indicates the 6th transmembrane helix.

B. Secondary structure prediction using DAS (www.sbc.su.se/~miklos/DAS/tmdas.cgi). The red arrow indicates the 6th transmembrane helix.
Reference: Cserzö M, Wallin E, Simon I, von Heijne G, Elofsson A (1997). Prediction of transmembrane α-helices in prokaryotic membrane proteins: the dense alignment surface method. *Protein Engineering* 10, 673-676.

C. Secondary structure prediction using HMMTOP (www.enzim.hu/hmmtop/server/hmmtop.cgi).
Reference: Tusnady GE, and Simon I (2001) The HMMTOP transmembrane topology prediction server. *Bioinformatics* 17, 849-850.

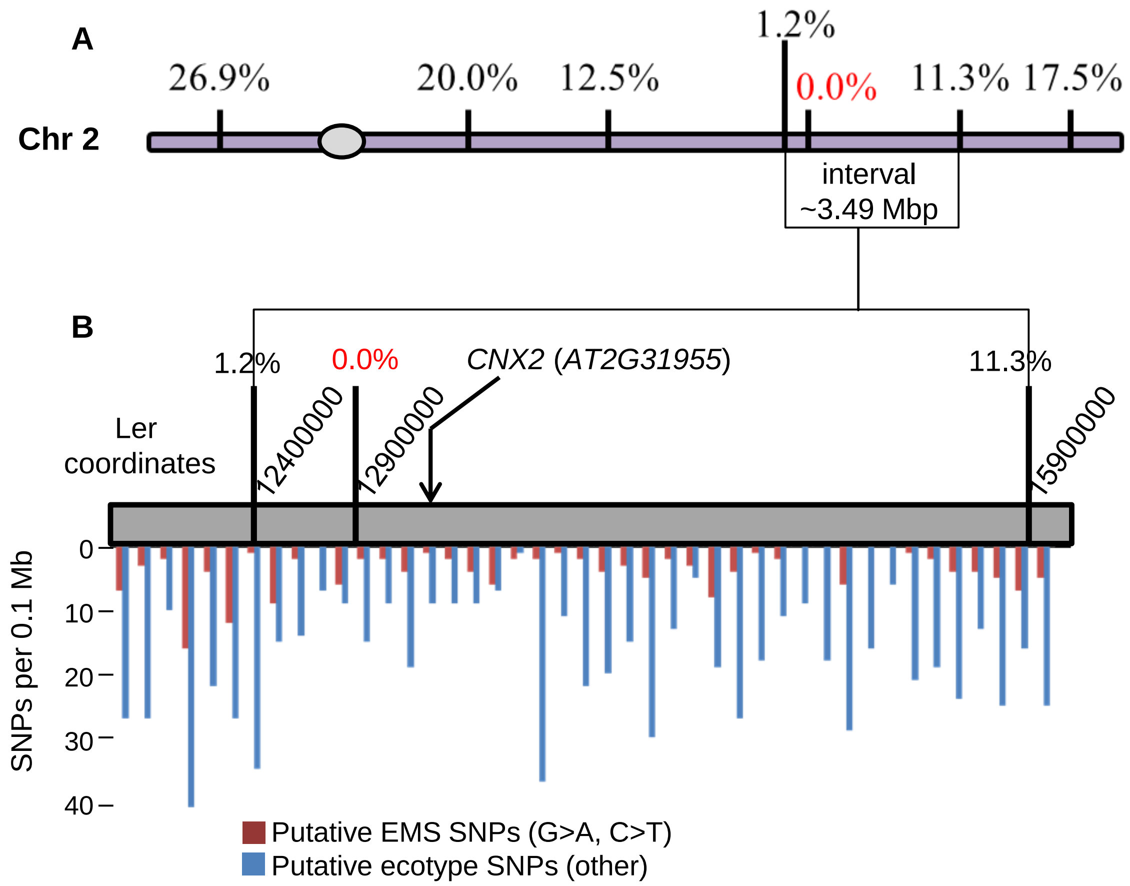

Supplementary Figure S3. Mapping-by-sequencing of the *xd22* mutation.
A. Diagram of chromosome 2 with the centromere (grey oval) and percentage recombination at specific SSPL markers. The percentages were calculated based on the analysis of 80 F2 plants from the *xd22* (Ler) x Col-0 with the *xd22* phenotype.
B. Frequency of SNPs in the mapping interval indicated in (A).

A

phenotype / line	n	Wild-type like [%]	Mutant-like [%]	Ungerminated [%]
Wild type	271	99.6	--	0.4
cnx2-1/CNX2	461	79.1	20.9	0.0

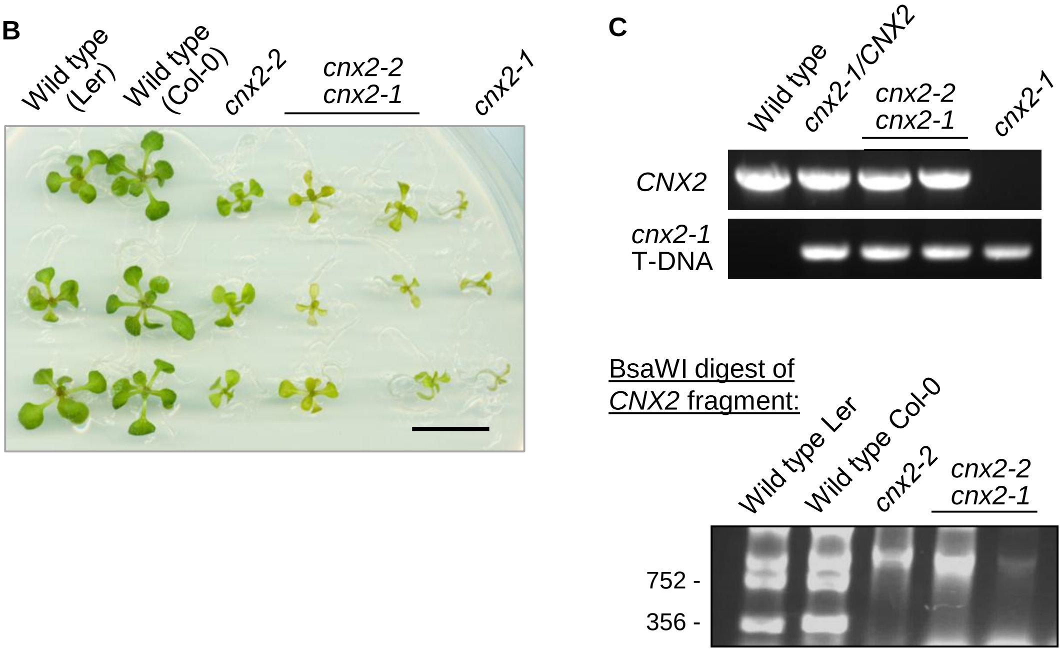

Supplementary Figure S4. Genetic analysis of the *cnx2-1* line.

A. Segregation frequency of wild-type and mutant phenotypes from a heterozygous *cnx2-1*/*CNX2* plant.

B. Allelism between *cnx2-2* and *cnx2-1*. Homozygous *cnx2-2* plants were cross-fertilised with heterozygous *cnx2-1/CNX2* plants and heterozygous *cnx2-2 cnx2-1* seedlings isolated from the F1 generation, and grown alongside *cnx2-1* and wild type parentals (Ler for *cnx2-2*, Col-0 for *cnx2-1*) on ½ MS plus 1% (w/v) sucrose. Scale bar is 1 cm.

C. PCR analysis and restriction digests to confirm the genotype of the seedlings shown in (B). The PCR product for *CNX2* is present in wild type and the *cnx2-2* allele (top panel), but the *cnx2-2* allele lacks a BsaWI restriction site (bottom panel), see Figure 4A for details.

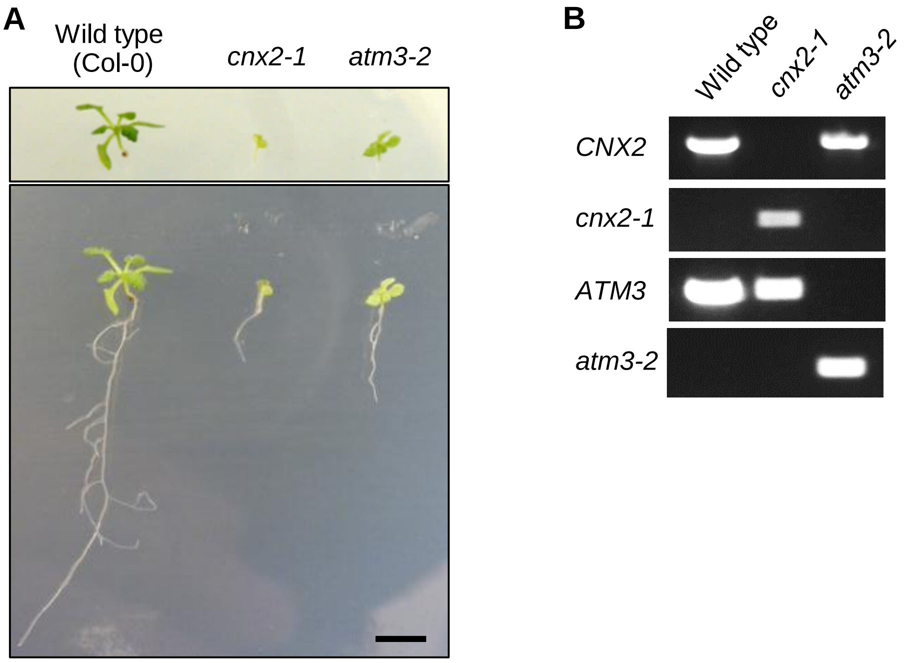

Supplementary Figure S5. Comparison of seedling growth in *cnx2* and *atm3* knockout mutants.

A. Growth of *cnx2-1* and *atm3-2* seedlings, knock-out mutants in *CNX2* and *ATM3*, respectively, on agar plates containing ½MS salts and 1% (w/v) sucrose for 3 weeks. Scale bar is 5 mm.

B. Genotype analysis of plants in (A). Primer pairs CNX2 2F and CNX2 2R were used to amplify the wild-type allele of *CNX2*; primers CNX2 2F and LBb1.3 were used to detect the T-DNA insertion in *CNX2* (*cnx2-1*). For the *ATM3* wild-type allele, primers ATM3 F1 and RWT were used, and primers ATM3 F1 and GK8409 for the T-DNA insertion in *ATM3* (*atm3-2*).

Table S1 List of oligonucleotides

**Oligonucleotide**	**5'-3' sequence**	**Use**
ACT2 F2	CCCAAAGGCCAACAGAGAGA	RT-PCR *ACTIN2*
ACT2 R2	ACCATCACCAGAATCCAGCA	RT-PCR *ACTIN2*
ATM3 RT-F1	TTGAGTGGTGGAGAGAAACAAA	RT-PCR *ATM3*
ATM3 RT-R1	TCCCGTTTTCCAGTACTACGAT	RT-PCR *ATM3*
CNX2 3F	GGGTGATCTTGCATTCGTTT	RT-PCR *CNX2*
CNX2 3R	CCGTCTGAAGGTGTGGAACT	RT-PCR *CNX2*
CNX2 2F	CTCAAGTTGGTTGCTTTTTCG	Genotyping c*nx2-1*
CNX2 2R	TTTGAAGTTCCCATCTGCAAG	Genotyping c*nx2-1*
LBb1.3	ATTTTGCCGATTTCGGAAC	Genotyping *cnx2-1*
CNX2endog 1F	AGAAACTAGATTTGCATACCTGGT	Genotyping *cnx2-2*
CNX2endog 1R	CGATTTCATAACCCTATCATGCCCTT	Genotyping *cnx2-2*
CNX2trans F	CGGTCTGGTGCTGATGATGA	Transgene *CNX2*
CNX2trans R	TAAAGTGCCCCTGATGATGAGATTTTCTTAAA	Transgene *CNX2*
ATM3 F1	GATGTCGAGAGGATCTCGATTCG	Genotyping *atm3-2*
ATM3 RWT	GAAAACTAGAGCTATTGAGAGTTACCA	Genotyping *atm3-2*
GK8409	ATATTGACCATCATACTCATTGC	Genotyping *atm3-2*
A1F	CTGGCGCGCCAAGCTTTTATTTCCTACTAACTAGTTAA	Cloning of *CNX2*
A1R	TCATCCTTGTAATCGACATTATATTGTTATGAATAAGC	Cloning of *CNX2*
At1g07810 F	GTTCACGGACAAAGAGCCTGAAAT	Mapping
At1g07810 R	AAGCAGTCAATATTGCAGGAAGGG	Mapping
At1g49610 F	ACATTTTCTCAATCCTTACTC	Mapping
At1g49610 R	GAGAGCTTCTTTATTTGTGAT	Mapping
At1g72650 F	TGTTTTTTAGGACAAATGGCG	Mapping
At1g72650 R	CTCCAGTTGGAAGCTAAAGGG	Mapping
At1g09940 F	TCATGACGTGAAGAAGAAGAAAA	Mapping
At1g09940 R	CATATCGCTGCTACTAATTTTAAACAA	Mapping
At2g04066 F	GGGATAATGGATAGGACTCACG	Mapping
At2g04066 R	GCTGAGAAGGCAAGGAAGAG	Mapping
At2g14890 F	GAAACTCAATGAAATCCACTT	Mapping
At2g14890 R	TGAACTTGTTGTGAGCTTTGA	Mapping
At2g39010 F	TCGTCTACTGCACTGCCG	Mapping
At2g39010 R	GAGGACATGTATAGGAGCCTCG	Mapping
AT2G21420 F	GATGCCTTTCTCCTGGTTG	Mapping
AT2G21420 R	AATATAGCCGTCGTCTTCATCA	Mapping
AT2G31070 F	AAAGAGATGAGAATTTGGAC	Mapping
AT2G31070 R	CATATCAATATATTAAAGTAGC	Mapping
AT2G44798 F	TGTTCTTCACTTTGCAAACCA	Mapping
AT2G44798 R	GTGGCAAATGGGCTAAACTA	Mapping
AT2G29995 F	CTGCATATTGTTAATGAGAAAAGAAT	Mapping
AT2G29995 R	TCATGTCGAAAACATATAATTGAGC	Mapping
At3g11220 F	GGATTAGATGGGGATTTCTGG	Mapping
At3g11220 R	TTGCTCGTATCAACACACAGg	Mapping
At3g26605 F	CCCCGAGTTGAGGTATT	Mapping

At3g26605 R	GAAGAAATTCCTAAAGCATTC	Mapping
At3g50820 F	GTTCATTAAACTTGCGTGTGT	Mapping
At3g50820 R	TACGGTCAGATTGAGTGATTC	Mapping
At4g01710 F	AGATTTACGTGGAAGCAAT	Mapping
At4g01710 R	GGTTAAAAATTAGGGTTACGA	Mapping
At4g10360 F	GCCAAACCCAAAATTGTAAAAC	Mapping
At4g10360 R	TAGAGGGAACAATCGGATGC	Mapping
At4g29860 F	GCCCAGAGGAAGAAGAGCAAACTAGC	Mapping
At4g29860 R	TGGGAATTCATGAGAGAATATGTGGGAC	Mapping
At5g22545 F	TAGTGAAACCTTTCTCAGAT	Mapping
At5g22545 R	TTATGTTTTCTTCAATCAGTT	Mapping
AT5G42600 F	CAGACGTATCAAATGACAAATG	Mapping
At5g42600 R	GACTACTGCTCAAACTATTCGG	Mapping
At5g63640 F	ATCACTGTTGTTTACCATTA	Mapping
At5g63640 R	GAGCATTTCACAGAGACG	Mapping
